# Antileishmanial Evaluation of the Leaf Latex of* Aloe macrocarpa*, Aloin A/B, and Its Semisynthetic Derivatives against Two* Leishmania* Species

**DOI:** 10.1155/2019/4736181

**Published:** 2019-02-24

**Authors:** Yitagesu Tewabe, Belete Kefarge, Habtamu Belay, Daniel Bisrat, Asrat Hailu, Kaleab Asres

**Affiliations:** ^1^Department of Pharmaceutical Chemistry and Pharmacognosy, School of Pharmacy, College of Health Sciences, Addis Ababa University, P.O. Box 1176, Addis Ababa, Ethiopia; ^2^Department of Materials Science and Engineering School of Mechanical, Chemical and Materials Science and Engineering, P.O. Box 1888, Adama, Ethiopia; ^3^Department of Microbiology, Immunology and Parasitology, Faculty of Medicine, College of Health Sciences, P.O. Box 1176, Addis Ababa, Ethiopia

## Abstract

The currently available antileishmanial drugs are either toxic or too expensive for routine use in developing countries where the disease is most common. Local people in the Somalia region of Ethiopia use the leaves of* Aloe macrocarpa* Todaro for the treatment of malaria, jaundice, and skin diseases. In our ongoing search for new, efficient, and safe antileishmanial drugs, we investigated the leaf latex of* Aloe macrocarpa* and its acid-hydrolyzed product aloin A/B (**1**), as well as the semisynthesized derivatives of aloin A/B, namely, aloe-emodin (**2**) and rhein (**3**) against promastigotes and axenically cultured amastigotes of* Leishmania aethiopica *and* L. donovani* clinical isolates. Activity study was carried out based on the fluorescence characteristic of resazurin added to drug-treated cultures. Oxidative hydrolysis of aloin A/B by ferric chloride and concentrated hydrochloric acid afforded aloe-emodin (**2**), which was further oxidized using sodium nitrite and concentrated sulfuric acid to furnish rhein (**3**). Cytotoxicity study of test substances was performed against human* monocytic* cell line* THP-1 using* Alamar Blue and cell viability was measured fluorometrically. The test compounds showed lower activity (IC_50_ = 6.7 to 12.1 *μ*M for promastigotes and IC_50_ = 3.6 to 10.2 *μ*M for axenic amastigotes) than the reference drug amphotericin B (IC_50_ = 1.3 to 2.7 *μ*M). However, amphotericin B (LC_50_ = 11.1 *μ*M) was much more toxic than the test compounds (LC_50_ = 369.2 – 611.6 *μ*M) towards human monocytic cell line (THP-1) despite its efficiency. As demonstrated in the current study, high selectivity indices (SIs) of the test compounds represent a remarkable advantage over the reference drug and highlight their potential use as templates for further development of safe leishmanicidal drugs.

## 1. Introduction

*Leishmania* is a protozoan parasite responsible for several pathologies known collectively as leishmaniases, causing disease ranging from skin lesions in cutaneous leishmaniasis to a progressive and fatal hepatosplenomegaly in visceral leishmaniasis [1]. Leishmaniasis is a widespread disease, affecting 12 million people worldwide with about 1-2 million estimated new cases occurring annually [2]. Although the disease is treatable, the lack of a vaccine, the adaptation of the vector and reservoirs to human environments, and the therapeutic failure have made control of the disease a difficult task [3]. In addition, there is no medication that is both completely safe and efficacious for all* Leishmania* and clinical manifestations [4, 5]. In the Americas, for over 6 decades, parenteral administration of the pentavalent antimonials (Sb-V) sodium stibogluconate and meglumine antimoniate has been used for treating all types of leishmaniasis [6]. In places where resistance to antimonials is common, other common chemotherapeutic treatments include amphotericin B and pentamidine [4].

In recent years, natural products have been explored as a good source of bioactive substances. Certain molecules isolated from natural sources represent a major breakthrough in the search for new antiprotozoal drugs, since there is urgent need for a new era of innovative medicines [7, 8].* Aloe macrocarpa *Todaro is a member of the group of aloes known as the “*saponaria*” group which have soft and spotted leaves, and a basal swelling of the perianth tube. The plants are clearly distinguished from the other Ethiopian members of the group by the numerous pale spots on the leaves, which also have distinct darker longitudinal lines, the perianth with a markedly globose basal swelling and a large capsule [9]. Local people in the Somalia region of Ethiopia use the leaves of this plant for the treatment of malaria, jaundice, and skin diseases.

TLC of the leaf latex of* A*.* macrocarpa* gave three major distinct spots, which were identified as aloin A/B (**1**) and its* p*-coumaroyl ester and* O*-rhamnoside derivatives, microdontin A/B, and aloinoside A/B, respectively. The identities of the compounds were confirmed by co-TLC with authentic reference compounds. In this paper, we discuss the antileishmanial activities of the leaf latex of* A*.* macrocarpa* and its major constituent aloin A/B (**1**) along with its semisynthetic derivatives aloe-emodin (**2**) and rhein (**3**).

## 2. Materials and Methods

### 2.1. General

Silica gel F_254_ (0.25 mm, Merck, Germany) was used for both analytical and preparative purposes. TLC spots were visualized by UV light of 254 and 366 nm. NMR spectra were recorded on a Bruker Avance DMX400, operating at 400 MHz for ^1^H and 100 MHz for ^13^C at room temperature using MeOH-*d*_4_ and DMSO-*d*_6_. ESI-mass spectra were recorded on an Ultimate 3000 LC-MS with the source voltage and temperature fixed at 3 kV and 250°C. All the chemicals were analytical grade and obtained from Pharmaceutical Fund and Supply Agency or from the Department of Pharmaceutical Chemistry and Pharmacognosy, School of Pharmacy, College of Health Sciences, Addis Ababa University.

### 2.2. Test Strains

*In vitro* antileishmanial activity tests were carried out against the promastigote stage and axenically cultured amastigotes of* L. aethiopica *and* L. donovani*. The two isolates were grown in tissue culture flasks containing RPMI 1640 medium supplemented with 10% heat-inactivated fetal calf serum, 100 IU penicillin/mL, and 100 *μ*g/mL streptomycin solution at 22°C for* L. aethiopica* and 24°C for* L. donovani*. Axenically cultured amastigotes were obtained from the late stationary phase promastigotes (3 × 10^6^ cells/ml). The cells were centrifuged and then resuspended in medium 199 with Hank's salts supplemented with 20% FBS, 2 mM L-glutamine, 50 IU/mL penicillin, and 50 *μ*g/mL streptomycin. The pH was adjusted to 5.5 using 1 N HCl. Following incubation of cells at 31°C for* L. aethiopica *and 37°C for* L. donovani* at 5% CO_2_, amastigote-like rounded morphology together with loss of flagella and cell clumping started appearing within 24 h. The parasites were kept for a week as some motile parasites with intermediate forms and short flagella were detected [10, 11]. The human monocytic leukemia cell line THP-1 cells were incubated in RPMI 1640 medium plus 10% hi-FCS and 20 ng/mL phorbol 12-myristate 13-acetate (PMA; Sigma) at 37°C and 5% CO_2_ for 72 h [12]. A cell-free medium was used to grow parasites* in vitro* and to set up the test system for determination of the IC_50_ values of the test substances.

### 2.3. Plant Material

Leaves of* A. macrocarpa* were collected from and around the town of Jigjiga, Somalia region, eastern Ethiopia. The identification and authenticity of the plant material was confirmed at the National Herbarium, Department of Biology (courtesy of Professor Sebsebe Demissew), Addis Ababa University, where a voucher specimen was deposited (JJU-YT 001).

### 2.4. Preparation of Latex and Semisynthesis

The latex of* A. macrocarpa* was collected as per the method described earlier [13]. Aloin A/B (**1**) and its derivatives aloe-emodin (**2**) and rhein (**3**) were prepared according to the procedure described by Tewabe et al. [13].

### 2.5. Acute Toxicity Study

Acute toxicity study was carried out as described earlier using the limit test dose of 2000 mg/kg according to OECD guideline for testing of chemicals on Swiss albino mice [14].

### 2.6. In Vitro Antileishmanial Assay

To a separate 96-well microtiter plate containing 100 *μ*L of complete culture medium, each of the test substances was added to triplicate wells to achieve a final concentration of 100 *μ*g/mL. Each one hundred microliter suspension of the parasites (3.5 × 10^6^ promastigotes or axenically cultured amastigotes /mL of* L. aethiopica *or* L. donovani*) obtained from the previous culture was then added to each well, and contents of each plates were maintained for 72 h, at room temperature for promastigotes stages of both strains, at 31°C and 37°C for axenically cultured amastigotes of* L. aethiopica *and* L. donovani*, respectively. After 68 h of incubation, 20 *μ*L (10% of the total volume of each well) resazurin (0.125 mg/mL) was added, covered with aluminum foil, and left at temperature mentioned above. Antileishmanial effect of the test substances was studied on the basis of fluorescence characteristic of resazurin added to drug-treated cultures [12, 15].

Antipromastigote effect was evaluated against clinical isolates of* L. aethiopica* and* L. donovani* strains, as per the method described by Abeje et al. [15].

### 2.7. Cytotoxicity Study of Test Substances in THP-1 Monocyte

THP-1 monocytes were plated onto 96-well plates at a density of 4 × 10^4^ cells per well (in 200 *μ*L volume) in the presence or absence of test substances and plates incubated at 37°C, 5% CO_2_ for 72 h. After adding Alamar Blue™ during the last 3 h of incubation, cell viability was measured fluorometrically as described above [12].

## 3. Data Analysis

Antileishmanial activity (IC_50_) was expressed as mean ± SD of triplicate measurements. IC_50_ values were evaluated from sigmoidal dose-response curves of percent inhibition using the computer software GraphPad Prism 8 (GraphPad Software, Inc., CA, USA), and values expressed as mean ± SD of triplicate experiments.

## 4. Results and Discussion

### 4.1. Semisynthesis

**Scheme 1:**
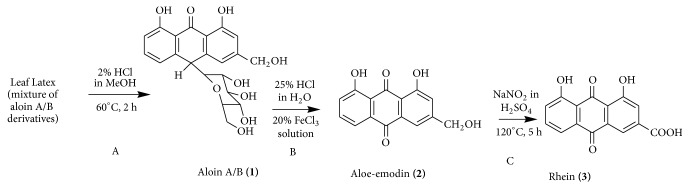
A: acid hydrolysis of components of the leaf latex of* Aloe macrocarpa* to aloin A/B; B: oxidative hydrolysis of aloin A/B (**1**) to aloe-emodin (**2**); C: further oxidation of aloe-emodin to rhein (**3**).

Acid hydrolysis of the latex of* A. macrocarpa* gave aloin A/B (**1**), which was used as a starting material for the synthesis of its derivatives. Aloin A/B was converted to aloe-emodin (**2**) by oxidative hydrolysis and further oxidation of the latter yielded rhein (**3**) (Scheme 1) [13]. The structures of all test compounds were unequivocally confirmed by comparing their spectral data (ESI-MS, ^1^H, and ^13^C NMR) with those reported in the literature [13].

### 4.2. Acute Toxicity Study

Acute toxicity study was carried out to investigate the toxicity profiles of the latex and aloin A/B isolated from the leaves of* A. macrocarpa *as well as those of the semisynthetic derivatives of aloin A/B (**1**), namely, aloe-emodin (**2**) and rhein (**3**). The results revealed that there is no significant sign of toxicity like increased motor activity, blinking eyes, tremors, convulsion, lacrimation, stimulation, muscle weakness, sedation, urination, salivation, diarrhea, lethargy, sleep, tremors, arching and rolling, and coma; and no death of mice was observed. However, minor signs of toxicity such as temporary hair erection and diarrhea were observed in two of the experimental animals. This indicates that the test substances are safe at the dose levels used, and their LD_50_ is above 2 g/kg as per the OECD guideline [14].

### 4.3. In Vitro Antileishmanial Assay

The currently available antileishmanial drugs have several limitations including high cost, poor compliance, low efficacy, and poor safety. Moreover, as most of these drugs were developed many years ago, resistant strains have emerged in recent years [16]. Thus, the severity of the disease and its high prevalence, particularly in developing countries, entail an urgent discovery of new drugs. Hence, this study was conducted with the aim of finding compounds having inhibitory effects on the* in vitro* growth of two* Leishmania* parasites, namely,* L. aethiopica *and* L. donovani*. Initially, the leaf latex of* A. macrocarpa* was tested for possible growth inhibitory effect against* L. aethiopica* and* L. donovani *promastigotes because of earlier reports that some plants belonging to the genus* Aloe* possess antiprotozoal activity [13, 15], and also that the plant is used traditionally for the treatment of malaria. Indeed, the leaf latex displayed potent inhibitory effect against the flagellated and extracellular promastigote stage of* L. aethiopica* and* L. donovani* with IC_50_ values of 1.90 and 1.92 *μ*g/mL, respectively. However, these results were considered preliminary as the promastigotes of* Leishmania* parasites are more prone to drug induced effect than the amastigotes [17]. Moreover, the clinical manifestations of the disease in humans are associated with the intracellular amastigotes, which are the developed forms of the parasite in vertebrate hosts, not the extracellular promastigotes. Therefore, the latex was further tested against the axenic amastigotes of the two pathogenic parasites. The tests showed that the latex has better activities than those observed against the promastigotes (IC_50_ = 1.60 and 1.74 *μ*g/mL, respectively).

In the present study aloin A/B, the major anthrone of the leaf latex exhibited a relatively weaker leishmanicidal activity than the latex on weight basis. These results were consistent with the previous report on the activity of aloin A/B against the promastigotes of* L. aethiopica* and* L. major* [15]. The* in vitro* antileishmanial effect of aloe-emodin, the oxidative hydrolysis product of aloin A/B, against the promastigote stage of the studied parasites was comparable to that of aloin A/B, whereas its potency was inferior to that of aloin A/B against the axenic amastigotes (Table 1).

Previously aloe-emodin was reported to have* in vitro* activity against* L. major *amastigotes with inhibitory concentrations of 40 *μ*g/mL [10]. However, no study was carried out on the effect of aloe-emodin against* L. aethiopica* and* L. donovani* promastigotes or axenic amastigotes. Rhein, the oxidized derivative of aloe-emodin, exhibited better potency than aloin A/B and aloe-emodin against both the promastigotes and amastigotes of the studied parasites. However, its potency against the promastigotes (IC_50_ = 6.7 and 7.3 *μ*M) and axenic amastigotes (IC_50_ = 3.6 and 4.1 *μ*M) of* L. aethiopica* and* L. donovani*, respectively, was lower than the reference drug amphotericin B (Tables 1 and 2). To the best of our knowledge, this is the first report that revealed the activity of rhein against* Leishmania* parasites.

From the data presented in Table 2, it appears that compounds having anthrone or anthraquinone moiety possess genuine leishmanicidal activity. According to a previous report [18], anthraquinones have the potential to intercalate DNA owing to their cyclic planar structure. Moreover, anthraquinones have been associated with a possible involvement in oxygen stress due to the presence of a quinone moiety in their structure [19]. Toxicity of quinones is mainly based on their ability to interact with essential nucleophiles in the cell, and also a consequence of their participation in redox reactions that can lead to the formation of deleterious reactive oxygen species (ROS) including  ^·^O_2_^−^, H_2_O_2_ resulting in oxidative stress [20]. The formation of ROS could probably explain the leishmanicidal activity of anthraquinone molecules. In view of the above, Schnur et al. [18] have proposed that anthraquinones could be used as leads for the development of antiprotozoal compounds. Other authors observed that several compounds having anticancer property possess antiprotozoal action and suggested that all anticancer natural products should be assessed for antiprotozoal activity [19]. In this respect, it is interesting to note that all the test compounds have been reported to have remarkable anticancer activities. Aloin A/B has been shown to induce cell cycle arrest and apoptosis in various human cancer cells, including breast [21], ovarian [22], uterine carcinoma [23], B16-F10 murine melanoma [24], and colorectal [25]. Similarly, aloe-emodin is endowed with selective* in vitro* and* in vivo* killing of neuroectodermal tumor cells [26], whilst rhein possesses* in vitro* activity against various cancers including nasopharyngeal carcinoma [27], tongue cancer [28], hepatocellular carcinoma [29], and lung cancer [30].

In order for a compound to be a candidate for antileishmanial drug, it should have potent leishmanicidal activity and low cytotoxicity. In the present study, cytotoxicity on human monocytic cell line (THP-1) was evaluated for all the test substances so as to determine their safety profile. As shown in Table 2, the cytotoxic effects (LC_50_) of the test compounds which ranged between 369.2 and 611.6 *μ*M on THP-1 cells were much higher than that of amphotericin B (LC_50_ = 11.1 *μ*M).

Selectivity index (SI) being an important tool to characterize the safety of biologically active compounds, the SI of each of the test substances as expressed by the ratio between cytotoxicity (LC_50_ value on THP-1 cells) and activity (IC_50_ value on* L. aethiopica* or* L. donovani* amastigotes), was determined. As shown in Table 3, all the test substances, particularly rhein, exhibited much higher SI than the reference drug amphotericin B, indicating their high selectivity towards the* Leishmania* parasites. Even aloin A/B, which was relatively less selective than the other test substances, was more than 5 fold as selective as amphotericin B. The results obtained in this study were consistent with other similar studies performed with different protocol for aloin A/B [15].

Although potency of the test substances against the intracellular amastigotes was lower than that observed for the reference drug, their low toxicity towards THP-1 cells and high SI provide a big advantage over the reference drug. Some authors claim that compounds having SI values greater than 20 are ideal candidates for further development of leishmanicidal drugs [31]. Based on this criterion, all the test compounds could be used as leads for development of safer and more potent antileishmanial drugs. Moreover, in view of the fact that the test compounds are active components of many traditional medicinal plants that have long been used as laxatives, they have an additional advantage of being used as clinical agents in their own right.

## 5. Conclusion

In conclusion, the results of this investigation revealed that the leaf latex of* A. macrocarpa*, aloin A/B, and its semisynthetic derivatives aloe-emodin and rhein has potent* in vitro* antileishmanial activities against* L. aethiopica *and* L. donovani* promastigotes and axenically cultured amastigotes. Although the potency of the test substances against the intracellular amastigotes is lower than that observed for the reference drug, their high selective toxicity towards the parasites ensures their potential to be used as a scaffold for the development of safe and cost effective antileishmanial drugs. To the best of our knowledge, this is in itself the first study that reports the leishmanicidal activity of the tested anthranoids against* L. aethiopica *and* L. donovani* amastigotes.

## Figures and Tables

**Table 1:** Antipromastigote activity of the test substances against *Leishmania aethiopica* and *Leishmania donovani*.

**Test substance**	Antipromastigote activity IC_50_ in *μ*g/mL^a^	
	*L. aethiopica*	*L. donovani*
Latex	1.90 ± 1.01	1.92 ± 0.42

Aloin A/B	3.94 ± 0.33 (9.4 *μ*M)	5.06 ± 0.22 (12.1 *μ*M)

Aloe-emodin	2.81 ± 0.43 (10.4 *μ*M)	2.85 ± 0.50 (10.6 *μ*M)

Rhein	1.91 ± 0.61 (6.7 *μ*M)	2.07 ± 0.11 (7.3 *μ*M)

Amphotericin B (reference)	2.43 ± 0.22 (2.6 *μ*M)	2.45 ± 0.21(2.7 *μ*M)

Media alone (NC)	0.00^b^	0.00^b^

1% DMSO (NC)	0.00^b^	0.00^b^

Values are expressed as mean ± SD; n = 3; NC: negative control; DMSO: dimethyl sulfoxide,

^a^Effective concentration required to achieve 50% growth inhibition in *μ*g/mL. ^b^No effect.

Values in parenthesis indicate concentration in micromolar (*μ*M).

**Table 2:** Effects of test substances on axenically cultured amastigotes of *Leishmania aethiopica* and *Leishmania donovani *and THP-1 monocytes.

**Test substance**	Antiaxenically cultured amastigotes activity IC_50_ in *μ*g/mL^a^		Cytotoxic effect in THP-1 LC_50_ in *μ*g/mL^b^
	*L. aethiopica*	*L. donovani*	
Latex	1.60 ± 0.01	1.74 ± 0.21	167.11 ± 0.42

Aloin A/B	2.80 ± 1.02 (6.7 *μ*M)	2.92 ± 0.15 (7.0 *μ*M)	154.32 ± 0.22 (369.2 *μ*M)

Aloe-emodin	2.23 ± 0.33 (8.3 *μ*M)	2.75 ± 0.03 (10.2 *μ*M)	145.45 ± 0.27 (538.7 *μ*M)

Rhein	1.01 ± 0.22 (3.6 *μ*M)	1.17 ± 0.42 (4.1 *μ*M)	173.70± 0.11 (611.6 *μ*M)

Amphotericin B (reference)	1.23 ± 0.41 (1.3 *μ*M)	1.55± 0.51(1.7 *μ*M)	10.23 ± 0.32 (11.1 *μ*M)

Media alone (NC)	0.00^c^	0.00^c^	0.00^c^

1% DMSO (NC)	0.00^c^	0.00^c^	0.00^c^

Values are expressed as mean ± SD; n = 3; NC: negative control; DMSO: dimethyl sulfoxide.

^a^Effective concentration required to achieve 50% growth inhibition in *μ*g/mL.

^b^The concentration that leads to death of 50% of THP-1 cells. ^c^No effect.

Values in parenthesis indicate concentration in micromolar (*μ*M).

**Table 3:** Selectivity indices of the test substances toward *Leishmania aethiopica* and *Leishmania donovani.*

**Test substance **	**Selectivity index**	
	***L. aethiopica***	***L. donovani***
Latex	104.44	96.04

Aloin A/B	55.11	52.84

Aloe-emodin	65.22	52.89

Rhein	171.98	148.46

Amphotericin B (reference)	8.31	6.60

Media alone (NC)	0.00	0.00

1% DMSO (NC)	0.00	0.00

NC: negative control.

## Data Availability

The data used to support the findings of this study are available from the corresponding author upon request.

## Abbreviations

CHCl_3_: Chloroform
DMSO-*d*_*6*_: Deuterated dimethyl sulfoxide
FeCl_3_: Ferric chloride
HCl: Hydrochloric acid
IC_50_: The half maximal inhibitory concentration
LR-ESIMS: Low resolution electrospray ionization mass spectrometry
MeOH-*d*_*4*_: Deuterated methanol
NMR: Nuclear magnetic resonance
OECD: Organisation for Economic Cooperation and Development
PTLC: Preparative thin layer chromatography
*R*_*f*_: Retention factor
SIs: Selectivity indices
THP1: Tamm-Horsfall Protein 1
TLC: Thin layer chromatography
UV: Ultraviolet.
